# Low use of artemisinin-based combination therapy for febrile children under five and barriers to correct fever management in Benin: a decade after WHO recommendation

**DOI:** 10.1186/s12889-018-5077-6

**Published:** 2018-01-22

**Authors:** B. G. Damien, B. Aguemon, D. Abdoulaye Alfa, D. Bocossa, A. Ogouyemi-Hounto, F. Remoue, J.-Y. Le Hesran

**Affiliations:** 1 Institut de Recherche pour le Développement (IRD), Maladies Infectieuses et Vecteurs: Ecologie, Génétique, Evolution et Contrôle (MIVEGEC), UMR IRD 224-CNRS 5290, University of Montpellier, Montpellier, France; 2 Centre de Recherche Entomologique de Cotonou, Bénin / Institut de Recherche pour le Développement, UMR 224-CNRS 5290 MIVEGEC, Cotonou, Bénin; 3 Département de Santé Publique, Faculté des Sciences de la Santé de Cotonou, Université d’Abomey-Calavi, Cotonou, Bénin; 4 Université Paris 8, UFR Etudes – Recherche – et Ingénierie en territoires – Environnements – Société, Saint-Denis, France; 5 Unité d’Enseignement et de Recherche en Parasitologie Mycologie/Faculté des Sciences de la Santé, Laboratoire du Centre de Lutte Intégrée contre le Paludisme, Université d’Abomey-Calavi, Cotonou, Bénin; 6 Institut de Recherche pour le Développement (IRD) / Mère et enfant face aux infections tropicales (MERIT), UMR 216, Cotonou, Bénin

**Keywords:** Care-seeking, ACT use, Behaviour change communication, Children under five years, Benin

## Abstract

**Background:**

Artemisinin-based combination therapy (ACT), used to treat uncomplicated malaria cases, is one of the main strategies of malaria control and elimination. One of the main objectives of the Benin National Malaria Control Program’s (NMCP) strategic plan is to ensure that at least 80% of uncomplicated malaria is treated with ACT within 24 h. Therefore, it was of great interest to measure whether the country case management of fever amongst children under five, adhered to the NMCP’s strategic plan and look into the barriers to the use of ACT.

**Methods:**

A cross-sectional survey based on a cluster and multi-stage sampling was conducted in two rural health districts in Benin. We recruited 768 and 594 children under five years were included in the northern and in the southern respectively. Data was collected on the general use of ACT and on the correct use of ACT that adheres to the NMCP’s strategy, as well as the barriers that prevent the proper management of fever amongst children. To assess the certain predictors of ACT usage, logistic regression was used, while taking into account the cluster random effect.

**Results:**

Among febrile children aged 6 to 59 months, 20.7% in the south and 33.9% in north received ACT. The correct use of ACT, was very low, 5.8% and in southern and 8.6% northern areas. Caregivers who received information on ACT were 3.13 time more likely in the south and 2.98 time more likely in the north to give ACT to their feverish child, PPR = 3.13[1.72–4.15] and PPR = 2.98 [2.72–3.11] respectively. Chloroquine and quinine, other malaria treatments not recommended by NMCP, were still being used in both areas: 12.3 and 3.3% in the south and 11.4 and 3.0% in the north.

**Conclusion:**

In Benin, the use and the correct use of ACT for febrile children remains low. The study also showed that having received information about the use of ACT is positively associated with the use of ACT. This point highlights the fact that efforts may not have been sufficiently integrated with social communication, which should be based on the behavioural determinants of populations.

**Electronic supplementary material:**

The online version of this article (10.1186/s12889-018-5077-6) contains supplementary material, which is available to authorized users.

## Background

According to the World Health Organization (WHO), children under five years of age are the most vulnerable group affected by malaria. In 2015, an estimated 429,000 malaria deaths occurred globally in the world and 70% of those deaths were by children under five. Amongst 23 African countries, a large proportion of febrile children (36%) are not taken to a health facility to receive care [1]. When prevention fails, effective malaria case management is the key for malaria control. Severe cases and death attributed to malaria can be avoided if families, especially caregivers, know the symptoms of malaria and can provide appropriate treatment as quickly as possible. Artemisinin-based combination therapy (ACT) is used for the treatment of malaria cases in areas where *Plasmodium* species are resistant to certain anti-malarial drugs. The decreasing cost of ACT, especially in the public sector with subsidies from the Global Fund (GF) and other funders, has led to its adoption by many endemic countries. In settings where parasitological diagnosis is not possible, anti-malarial treatment must be provided based on the likelihood that the illness is malaria [2]. A large proportion of fever cases, especially in Africa, are treated as cases of malaria without parasitological confirmation (50 to 90%) [1, 3–5] and most febrile children under five years of age are taken care of at home [6]. Care-seeking at health centres (HC) was not immediate and typically occurred with delay [7]. In certain sub-Saharan countries, Integrated Management of Childhood Illness (IMCI) was introduced into the community case management of fever instead of treatment at HC. The IMCI strategy focuses on malaria, diarrhoea, pneumonia, and malnutrition diagnosis and treatment [8]. This approach requires malaria parasitological diagnosis before treatment with ACT [9].

In Benin, *Plasmodium falciparum* is the predominant species of malaria, more than 90% of infection [10]. Every year, roughly 40% of malaria cases in HC occur amongst children under the age of five [11]. In 2005, a large decrease in the therapeutic efficacy of chloroquine (CQ) and sulfadoxin-pyrimetamine (SP) was documented at 86 and 50% [12]. This was confirmed in 2011 by the high level of CQ and SP resistant molecular markers found amongst Beninese children [13]. Since 2004, the National Malaria Control Programme (NMCP) has encouraged the use of ACT to treat uncomplicated malaria cases [14]. However, as described in Kenya [15], the use of ACT in the behaviour of caregivers is linked to many factors like (e.g. lack of money to buy it, distance between home and the HC *etc*). These factors were also similar for medical practitioners who did not prescribe ACT according to the policy [14, 16, 17]. In both rural and urban areas of Benin*,* self-medication is highly prevalent following familial practice and advice from friends/neighbours [18, 19]. From 2011 to 2012, findings from the Benin Demographic and Health Survey (DHS) showed that the use of health services by the population remained low, at about 44%. The usage of health services was especially low amongst febrile children, where only 39% sought care at a HC. The DHS noted similarly low care-seeking for acute pneumonia and diarrhoea (31 and 37% respectively) amongst children under five [20].

From 2007 to 2011, the IMCI was implemented at the community level, where no access to parasitological diagnosis of malaria was available. Community Health Workers (CHW), individuals or groups, could sell ACT treatment, but only for febrile children under five [16]. At this time, ACT was also sold at health facilities. The CHW are usually recruited and trained during development projects carried out by national or international Non-Government Organizations (NGOs) working under the coordination and supervision of the Ministry of Health. From 2006 to 2010, one of the main objectives of the NMCP strategic plan [21] was to ensure that at least 80% of uncomplicated malaria cases amongst children under five were treated within 24 h of symptom onset. This objective was created because the use of ACT is a considerable step to malaria elimination. There was a great interest in understanding the barriers to fever management and the appropriate use of ACT amongst children under five in Benin. The objective of our study was to measure the use and the the NMCP’s defined correct use of ACT and determine the barriers to properly treating fever amongst children under five.

## Methods

### Study setting

This study was carried out in two rural health districts in Benin, from April to May 2011 in Ouidah-Kpomassè-Tori Bossito (OKT) in the south and from July to August 2011 in Djougou-Copargo-Ouaké (DCO) in the north. The OKT area is situated at 50 km west from Cotonou and the DCO area at 381 km from Cotonou (Additional file 1). In 2013, the OKT and DCO areas had populations of 286,711 and 411,835 respectively [22]. Children under five years were roughly 17% of the total population [20].

In the OKT district, there are two rainy seasons (April to July and September to November). In this area, malaria is mesoendemic, with a 35% prevalence rate of *P. falciparum* infection among children aged 0–5 years [10, 23]. Malaria transmission is also perennial with no seasonal variation [23].

In the DCO district, there is only one rainy season (May to November) and the prevalence of *P. falciparum* infection among children aged 0–5 years old is high at > 75% [23, 24]. A study conducted in north-west Benin showed the existence of malaria cases in the dry season [25] proving that malaria transmission is perennial in northern Benin as well.

The characteristics of malaria transmission in these areas were described previously in several epidemiological studies [10, 23, 25, 26].

Each commune in Benin has a HC [17]. ACTs were available in the public sector in HC, and with CHW. ACT was sold (as per public policy) to CHWs at a cost of 150 XOF (USD $.0.25; 6 tablets per blister pack) for children aged < 36 months old and 300 XOF (USD $.0.50; 12 tablets per blister pack) for children aged 37–59 months old. During the study, there was no rapid diagnosis test at a community level.

### Behaviour change communication intervention in study areas

In March 1999, the Benin Ministry of Health (MoH) formally adopted the IMCI strategy [27] and since 2006, the National Communication Plan on malaria was also developed [28]. In areas of the study (OKT and DCO), the IMCI and malaria prevention strategy included behaviour change communication (BCC) were implemented three years before the study, as well as during it.

In the OKT area, the Palu Alafia project funded by GF in 2008, had implemented the Community-based IMCI (C-IMCI) and malaria prevention strategy. These interventions were implemented in several villages located five kilometres from the HC.

In the DCO area, three projects from three different organizations were involved in febrile case management at the community level amongst children under five years old. Their two main activities were malaria case management and prevention using the BCC strategy by CHW. The "Projet d’appui au Renforcement des Départements des Zones Sanitaires" (PARZS) project (2010–2014) funded by Cooperation Technique Belge (CTB) [29] and Basic Support for Institutionalizing Child Survival-III (BASICS III) project funded from 2008 to 2011 by the US Agency for International Development (USAID) [30] worked closely with the MoH and focused on C-IMCI (malaria, diarrhoea and cough). Africare, an NGO, and sub-recipient of GF, worked on the subject of malaria treatment with ACT from 2011 to 2013.

In both areas, educational meetings (discussions with small groups of mothers) and home visits to mothers were used by CHW to implement their BCC strategy and promote the use of ACT. For this purpose, training images, HC meetings, radio, etc. were used as channels of communication. The contents of the BCC information were based on the NMCP national communication plan on malaria prevention and treatment updated in 2006 [21].

### Design and sampling

The study was a cross-sectional household survey with a multi-stage sample as recommended by WHO where a sampling frame is not available [31]. A list of villages was extracted from the Institut Nationale de la Statistique Appliquée à l’Economie (INSAE) data base and a three-degree stage sampling procedure was used: village, household and child. In the OKT area, 137 villages were identified encompassing 48,538 households and 45,264 children under five. In the DCO area, 136 villages were identified containing a total of 37,178 household and 57,284 children under five. We assumed that around 25% of the total of the villages per study area. Thus, 35 villages were randomly selected in each health district to participate in this study. A cluster was defined as village of residence.

### Household inclusion criteria

For a household to be included in this survey the primary caregiver was required to be at home and at least one febrile child had to have been registered in the study.

### Children inclusion criteria

The children who met the criteria below were enrolled in the study: (i) aged 0 to 59 months (ii) history of fever within the past two weeks (iii) no signs of severe malaria during the febrile episodes (e.g. convulsion, unconsciousness, severe anemia). (iv) written informed consent from parents. If more than one child in the household fit the inclusion criteria, one child was selected at random.

### Sample size estimation

We assumed that the number of fever cases attributable to malaria was 33% [10] in southern Benin and 22.2% in northern [32]. The calculation of the sample size was based on a statistical power of 80%, considering the estimated cluster effect, k = 2. When additionally, 10% of cases was considered, it was necessary to recruit 772 children in the south (22 children per cluster), and 595 children in the north (17 children per cluster).

### Data collection

Data collection teams and supervisors received a six-day training focused on the administration of the questionnaire and sampling procedures. Two teams of field surveyors were created per study zone. The local language varied between the northern and southern regions therefore different data collection teams were used for each region. They were trained to use pictures showing ACT boxes or packaging to interview mothers or caregivers about ACT usage during cases of fever. The pictures included the types of ACT commonly used in Benin, ACT deployed by NMCP in the study area. After obtaining written informed consent, a questionnaire was asked to the mother or guardian of the child included in the study. All questionnaires were reviewed by the supervising team.

During the interviews, detailed socio-demographic information of the mother or caregiver (age, school level, occupation) was collected. In addition, the size of the household, information about the febrile children in the household (age, sex, and rank number of the siblings) as well as the medical treatment the children received was all noted. Images of ACT boxes were shown to the caregivers to help identify which type of treatment was given to the children. The field investigators also observed if ACT, or empty boxes, were found within the household demonstrating that caregivers were aware of the treatment.

The use of ACT, the source and time of care, and the use of thermometer were all topics investigated. Questions were also asked about medication typically used when a fever is present. The reasons justifying non-use of ACT was also collected.

Each day, the supervisors verified the presence of surveyors on the field and completed questionnaires were validated at the end of the day.

### Definition of indicators

The “use of ACT” was calculated as the proportion of febrile children who received ACT when a fever was present. This calculation was done 24 and 48 h after the onset of fever.

A composite measure, based on two criteria, was developed to assess the correct use of ACT: i) appropriate number of ACT tablets or spoonfuls given based on a child’s age as per national malaria treatment guidelines [21], and ii) the frequency per day and duration of treatment (i.e. two doses per day for three days). Any use of ACT that did not meet these criteria was considered inappropriate.

### Data management and analysis

All collected data was entered and checked for errors using the Access 2003 program while a statistical analysis was completed using SAS version 9.3 (SAS Institute Inc., Cary, NC, USA).

An analysis was done in each area to understand the specific issues of malaria control in each endemic region in the hope of providing an adequate local solution.

Median and quartiles were calculated for continuous variables and percentages for categorical variables. Social and demographic characteristics were described for mothers and children. Knowing that children received ACT after six months of age, the type of ACT usage (use and correct use) was calculated in this age group.

Logistic regression, taking into account the cluster random effect, was used to test an association between i) characteristic of mother and child and ii) use of ACT in febrile children. Prevalence Risk Ratios (PRRs) were used as a measure of association because the outcome (use of ACT) was higher than 5%. When the outcome is higher than 5%, the more precise the PRR is [33]. For multivariate analysis, a logistic regression models were used to determine the adjusted PRRs and their associated 95% confidence intervals. Multivariable models were adjusted for potential confounders, which were added if they showed statistical significance of 0.25 or less at bivariate analysis. Confounding factors stayed in the model if they had been found to be significantly associated with the use of ACT.

### Ethical approval

This study followed ethical principles recommended by the Edinburgh revision of the Helsinki Declaration. Ethical clearance for this study was obtained from the National Ethical Committee for Medical Research in Benin (CNERS, Number 003, 24 March 2011, Institutional Review Board No. 00006860) and Institut de Recherche pour le Développement (IRD)‘s consultative committee for deontology and ethics (CCDE).

## Results

### Socio-demographic characteristics of children under five with fever and their mothers or caregivers

In a household with multiple febrile children, a random selection of one child per household was done 20 times in the south and 52 times in the north.

**Fig. 1:**
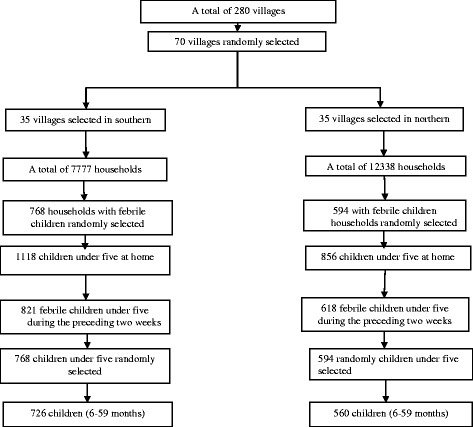
Study profile. Only febrile children aged (6–59 months) was used to determine factors associated to the use of ACT

In the OKT district, a total of 768 febrile children were selected (Fig. 1) with a median age of 25.5 months (Q1 = 13.0; Q3 = 36.5) and a sex ratio M/F of 1.1. The median age of caregivers (mother or guardian) was 29 years old (Q1 = 25.0, Q3 = 35.0). The majority (62.0%) of the caregivers never went to school and their main economic activity was trade and business (74.4%), (Table 1).

**Table 1:** Characteristics of children and caregivers, according to study areas, OKT and DCO health districts, Benin, 2011

	OKT (South)		DCO (North)	
Characteristics	n	%	n	%
Children				
Age (months)				
0–23	417	54.3	291	49.0
24–60	351	45.7	303	51.0
Sex				
Male	397	51.7	307	51.7
Female	371	48.3	287	48.3
Mother or caregiver’s (years)				
Age				
≤ 24	165	21.5	140	23.6
25–29	239	31.1	128	21.6
30–34	179	23.3	160	26.9
≥ 35	185	24.1	166	27.9
School level				
None	473	62.0	450	75.8
Primary school at least	295	38.0	144	24.2
Profit- making activity				
Farmer	184	24.0	149	25.1
Trade/business	572	74.4	377	63.5
House wife	12	1.6	68	11.4

The table contains two parts: information about the children and information about mothers or caregivers. The title of each part is highlighted by using the bold text

In the DCO district, 594 febrile children were selected (Fig. 1), with a median age of 24.0 months (Q1 = 14.5; Q3 = 36.0) and a sex ratio M/F of 1.1. The caregiver’s median age was 30 years old (Q1 = 25.0; Q3 = 35.5). The majority of them never went to school (75.8%) and their main economic activity was crafts (63.5%), (Table 1).

### Initial source and time of care

#### OKT district

Among 768 febrile children aged 0 to 59 months, 766 sought care. 97.0% (745/768) of caregivers’ initial source of care was at home while 23.4% (180/768) of them went to a HC after first treating their children at home and 1% (7/768) went directly to the CHW. Only 18.0% (137/768) of them had their body temperature taken by a thermometer. A proportion of 63.8% of children received initial treatment within 24 h of having a fever and 80.5% within 48 h.

#### DCO district

Among 594 children aged 0 to 59 months, 98.3% (585/595) initially received care at home. Body temperature taken by a thermometer at home or at HC was used to confirm fever for 37.8% (225/594) of children. A proportion of 34.8% (207/594) went to a HC after the first home treatment and 10% (55/594) went directly to the CHW. 61.7% received initial treatment within 24 h of the onset of fever and 91.6% within 48 h.

### Medicines used by caregivers for children

In the OKT district, a large percentage of children were initially treated with herbal tea (37.5%), anti-malarial drugs (40.6%) or paracetamol (13.0%). A combination of herbal tea plus paracetamol (8.9%) and anti-malarial plus paracetamol (13.0%) - were also used. In the DCO district, anti-malarial drugs (46.6%), herbal tea (24.9%), paracetamol (15.3%), individually, and anti-malarial plus paracetamol (24.5%) were used as the first treatment for febrile children. The commonly used anti-malarial drugs were ACT, CQ, and quinine. The use of CQ was 12.3% in the south and 11.4% in the north while the use of quinine was 3.3% in the south and 3.0% in the north (Table 2). No case of ingestion of artesunate/artemether in monotherapy was observed in either area.

**Table 2:** Drugs used by caregiver’s for the management of fever, OKT and DCO health districts, Benin 2011

		OKT (South)	DCO (North)
Variables		n (%)	n (%)
Anti-malarial alone		92 (12.0)	131 (22.0)
	ACT	58 (7.6)	106 (17.8)
	Chloroquine	21 (2.7)	18 (3.0)
	Quinine	11 (1.4)	6 (1.0)
	Amodiaquine	1 (0.1)	1 (0.2)
	Sulfadoxine-pyriméthamine	1 (0.1)	
Paracetamol alone		62 (8.1)	91 (15.3)
Aspirine alone		7 (0.9)	9 (1.5)
Herbal tea alone		287 (37.5)	148 (24.9)
Anti-malarial + paracetamol		99 (13.0)	146 (24.5)
	ACT	49 (6.4)	84 (14.1)
	Chloroquine	43 (5.6)	50 (8.4)
	Quinine	7 (1.0)	12 (2.0)
Anti-malarial+Herbal tea		84 (11.0)	
	ACT	47 (6.1)	
	Chloroquine	30 (3.9)	
	Quinine	7 (0.9)	
Paracetamol + Herbal tea		68 (8.9)	51 (8.6)
Anti-malarial+ Herbal tea + paracetamol		35 (4.6)	
Others (antibiotoics, iron, anti-helminths)		34 (4.4)	18 (3.0)

### Use of ACT associated with communication strategy

#### OKT district

Seven hundred twenty six febrile children aged 6 to 59 months were included in this the study. Among them 20.7% (CI 95%: 17.7–23.6) received ACT drugs. The proportion of caregivers who sought care for their child and gave ACT within 24 h and 48 h was 10.6% and 14.9% respectively.

86.9% of caregivers stated to have received information at least once about ACT use. The source of information was from HC (34.0%), CHW (49.9%), radio/television (7.9%) combined or not with mass sensitization (12.8%).

In a univariate analysis, “received information about ACT” and “confirmed fever by thermometer” were associated with the use of ACT for febrile children by the mothers or caregivers, PRR = 1.84 [CI95%: 1.33–2.41], and PRR = 3.10 [CI95%: 1.71–4.12] respectively. The factors not significantly associated with the use of ACT were the age and sex of the children, age, school level and occupation of mother or caregiver, the size of the household, number of children and the number of present children under five (Table 3).

**Table 3:** Factors associated with the use of ACT, OKT health district, south of Benin, 2011

Variables	Use of ACT			
	N	%	Crude PRR (95% CI)	Adjusted PRR (95% CI)
Information about children				
Age group (month)				
0–23	375	51.65	1	
24–60	351	48.35	1.10 [0.83–1.42]	
Sex				
Male	381	52.48	1	
Female	345	47.52	1.05 [0.76–1.40]	
Rank number in the siblings				
≤ 2	268	39.91	1	–
> 2	458	63.09	0.80 [0.59–1.08]	–
Use of thermometer				
No	596	82.09	1	1
Yes	130	17.91	1.84 [1.33–2.41]^a^	1.84 [1.31–2.45]^a^
Number of children aged 0–5 years in the household				
≤ 1	429	59.09	1	–
> 1	297	40.91	0.91[0.64–1.26]	–
Information about mothers or caregivers				
Age (year)				
≤ 24	149	20.52	1	–
25–29	225	30.99	0.96 [0.65–1.37]	–
30–34	173	23.83	0.94 [0.61–1.38]	–
≥ 35	179	24.66	0.83 [0.54–1.23]	–
Primary school at least				
No	447	61.57	1	–
Yes	279	38.43	1.01 [0.74–1.35]	–
Occupation				
Farmer	265	36.50	1	–
Trade/business	441	60.74	1.11 [0.79–1.50]	–
House wife	20	2.75	0.51 [0.12–1.69]	–
Get information about the use of ACT				
No	95	13.09	1	1
Yes	631	86.91	3.10 [1.71–4.12]^a^	3.13 [1.72–4.15]^a^

The table contains two parts: information about the children and information about mothers or caregivers. The title of each part is highlighted by using the bold text

The variable “Get information about the use of ACT” was significantly associated to the use of ACT adjusted on the “Use of thermometer”

^a^Statistically significant value of Prevalence Ratio Risk (PRR)

In a multivariate analysis, the responses “received information about ACT” and “confirmed fever by thermometer”, were significantly associated with the use of ACT, PRR = 3.13 [CI95%: 1.72–4.15], (Table 3).

#### DCO district

Among 560 children aged 6 to 59 months, 33.9% (190/560) received ACT treatment during febrile episodes. The proportion of caregivers who sought care for their child and administered ACT within 24 h and 48 h was 19.5 and 28.8% respectively.

About 55.0% (308/560) of mothers or caregivers declared having received information on the use of ACT at least once. The sources of information were HC (38.6%), CHW (31.6%), radio/television (1.1%), combined or not with mass sensitization and masse sensitization (20.4%).

In a univariate analysis, the responses “Age of children”, "Receive information about ACT by caregiver" and “school level of caregiver” were significantly associated with the use of ACT. Especially, the use of ACT was significantly higher in the group of children ≤23 months old compared to the group > 23 months old: PRR = 0.61 [CI95%: 0.39–0.93]. “Received information about ACT”, and "have at least primary school level", were positively associated with the use of ACT, PRR = 2.95 [CI95%: 2.70–3.09], and PRR = 1.35 [CI95%: 1.02–1.70] respectively. The sex of children, age and occupation of mother, size of household, number of children and number of children under five years lived in the household were not significantly associated with the use of ACT by febrile children (Table 4).

**Table 4:** Factors associated with the use of ACT, DCO health district, north of Benin, 2011

Variables	Use of ACT			
	N	%	Crude PPR (95% CI)	Adjusted PPR (95% CI)
Information about children				
Age group (month)				
0–23	257	45.89	1	1
24–60	303	54.11	0.61 [0.39–0.93]^a^	0.58 [0.34–0.94]^a^
Sex				
Male	288	51.43	1	
Female	272	48.57	1.13 [0.90–1.39]	
Rank number in the siblings				
≤ 2	220	39.29	1	–
> 2	340	60.75	1.07 [0.90–1.47]	–
Use of thermometer				
No	350	62.50	1	–
Yes	210	37.50	0.77 [0.49–1.14]	–
Number of children aged 0–5 years in the household				
≤ 1	341	60.89	1	–
> 1	219	39.11	1.18 [0.87–1.52]	–
Information about mother or caregiver				
Age (year)				
≤ 24	126	20.50	1	–
25–29	118	21.07	1.18 [0.78–1.31]	–
30–34	155	27.68	1.28 [0.86–1.32]	–
≥ 35	161	28.75	1.00 [0.62–1.49	–
Primary school at least				
No	424	75.71	1	
Yes	136	24.29	1.35 [1.02–1.70]^a^	–
Occupation				
Farmer	243	43.39	1	
Trade/business	249	44.46	0.78 [0.52–1.11]	–
House wife	68	12.14	0.89 [0.49–1.43]	–
Get information about the use of ACT				
No	251	44.82	1	1
Yes	309	55.18	2.95 [2.70–3.09]^a^	2.98 [2.72–3.11]^a^

The table contains two parts: information about the children and information about mothers or caregivers. The title of each part is highlighted by using the bold text

The variable “Get information about the use of ACT” was significantly associated to the use of ACT adjusted on the children age group

^a^Statistically significant value of Prevalence Ratio Risk (PRR)

In a multivariate analysis, the responses “Received information about ACT” and “Confirmed fever by thermometer” were significantly associated with the use of ACT for febrile children by mothers or caregivers, PRR = 2.98 [CI95%: 2.72–3.11], (Table 4).

### Correct use of ACT based on NMCP/WHO recommended treatment

In the OKT district, 10.6% of febrile children were correctly treated: 5.8% after 24 h and 8.6% after 48 h. Artemeter-lumefantrine (AL) was the most commonly type of ACT used (98.0%). Four mothers or guardians declared that they had given ACT drugs to children younger than six months.

In the DCO district, 13.9% of febrile children were correctly treated: 8.6% after 24 h and 12.7% after 48 h. All children aged six to 59 months were treated with AL, while no children younger than 6 months were treated with ACT.

### Reasons to justify the non-use of ACT

Various reasons were given by caregivers to justify the non-use of ACT for febrile episodes. In some cases, caregivers gave more than one response. Among 631 total responses in the OKT district, 23.0% declared "Don’t know that ACT can be used", 18.0% declared "Unavailability of ACT in HC, and with CHW", 9.0% declared "Preference to use paracetamol instead of ACT", 9.0% responded "Prescription of another anti-malarial drug in the HC", and 10.0% declared “absence of serious fever” (Additional file 2).

In the DCO district, among 400 total responses, 54.5% responded "Do not know if ACT can be used", 19.0% "Prescription of another anti-malarial drug at the HC", 11.5% “Lack of funds”, 5.0% "Unavailability of ACT in HC, and with CHW", 2.0% "Preference to use paracetamol instead of ACT", and 2.0% of “Absence of serious fever” (Additional file 2).

### Source of ACT

In the OKT district, ACT was bought mostly from CHW (58.7%) and HC (34.7%). The main reason of this was the availability of drugs in this area (53.3%). In the DCO district, ACT was bought mostly from CHW (58.5%) and HC (40.8%). The source of ACT depended on i) the distance between their household and the availability of ACT 21.3% (35/164), ii) the prescription of ACT on the source 42.7% (70/164), and iii) the recommendation of this source during mass sensitization efforts 31.1% (51/164).

## Discussion

In this study, we measured the use of ACT and its associated factors among febrile children younger than five years old. We also investigated the correct use of ACT based on NMCP/WHO recommended treatment, the anti-malarial drugs commonly used, the sale agency of ACT and the declared reason of the non-use of ACT.

The main findings of the study showed that, based on the testimonies of mothers or caregivers, the use of ACT in febrile children in Benin is lower than expected. Among children aged 6 to 59 months, 20.7 and 33.9% received ACT in the south and in the north respectively. The correct use of ACT within 24 h as recommended by NMCP/WHO was at a low of 5.8% in the south and 8.6% in the north.

### Use of ACT

To overcome the challenges of anti-malarial drug resistance and improve treatment efficacy, WHO expert panels have recommended since 2001 the use of a fixed-dose of ACT as first-line treatment for uncomplicated *P. falciparum* malaria [34, 35].

From 2008 to 2013, contrary to policy, CHW service was used to treat febrile cases with ACT without any parasitological confirmation. This practice can lead to inadequate community-based malaria services for children under five years.

While ACT has been a national policy in Benin since 2004, treatment with ACT remains far from universal. This treatment was already shown to be poorly used in 2006 in Benin. The use of ACT for febrile children under five years was less than 1% [36]. Descriptive statistics using data from 12 Demographic and Health Surveys in 2010–2012 reported the prevalence of fever, diagnostic tests, medicine use, and socio-economic states which showed that the use of ACT was similar to another study conducted in the south of Benin during the same year with, 17.5% (14.0–21.0) of use [37]. Results of ACT use among febrile children aged under five from Ghana (69.1%) between 2009 and 2011 [38], from Uganda (44%) in 2012 [39], and from Tanzania (34.2%) among patients aged three to 12 years old in 2010 [40] were higher than those observed in Benin. In Ghana and in Benin, ACT was not free during the period of the studies according to national policy. The practice of self-medication using medicines more readily available and less expensive as first treatment for fever, could explain why the use of ACT was so weak in Benin.

#### Correct use of ACT

In our study, the prevalence of ACT correct use within 24 h according to NMCP/WHO was less than 10% in both study areas. In Uganda, the use of ACT within a day after the onset of fever was 30–36% in 2011–2012 [39, 41] after Affordable Medicines Facility – malaria (AMFm) programme which has since been integrated into the Global Fund core grant management and financial processes [42]. For most malaria endemic countries, the data on the correct use of ACT is not available. This question was investigated by the present study and showed that the three-day treatment of ACT is typically done at home. When CHWs provide ACT medication, only the first dose is supposed to be observed [28]. Therefore, it seems that there is no standard on the correct use of ACT by caregivers. It may not be clear enough to caregivers that they should respect the dosage and duration of ACT treatment. For example, for each age or weight, a fixed dose (six or 12 tablets) of ACT is given to the caregivers. When children vomited or spit up tablets at home, there was not more medication available to complete the dosage. Furthermore, the use of spoons to administer drugs to young children is not yet studied enough in rural areas. It suggests that caregivers and CHW need more training in order to increase the correct administration of ACT. More operational research is also necessary to understand how the tablets of ACT are being used in rural areas.

#### Other medications

It was not surprising that herbal tea was often used as treatment in the areas of study. There is a longstanding cultural practice of using herbal teas to treat illness [7, 19]. These herbal teas are widely accessible, and also culturally accepted by caregivers. The use of non-recommended modern medicines (paracetamol, chloloquine, quinine) alone or combined with another treatment was also very common. Paracetamol, especially popular, is an antipyretic, inexpensive, highly effective in decreasing fever, and easy to buy in informal structures like local markets [43–45]. The quinine drug is often perceived as the most effective treatment of malaria as it is recommended for severe malaria cases therefore, in cases of uncomplicated malaria, people seem to prefer the use of oral or intravenous quinine. In the Republic Democratic of Congo, self-medication with quinine to treat malaria was found in 20% of primary-school students in 2008 [46]. Another hypothesis to explain the continued usage of CQ and quinine instead of ACT can be attributed to the limited availability of ACT, especially stock-outs with formal distributors [47–49]. A study review showed that in the absence of ACT, quinine and CQ were prescribed [50]. The low cost of CQ and quinine which were as cheap as ACT (around 100 FCFA per 10 tablets), was favourable to the use of this anti-malarial treatment. CQ was also used for a long time as an anti-malarial drug in Benin for children and pregnant women [51] and this past policy could be influencing this population’s present behaviour.

In Benin, the high level of *P. falciparum* resistance to CQ and SP should dissuade the use of these drugs for malaria cases treatment in the country [13]. As in Benin, CQ is still used in Ghana [52]. In fact, the traffic and use of drugs in Benin, especially for malaria treatment is not well controlled [53, 54]. More policies and the application of policies already in place should be implemented to obtain better management of drugs in Benin.

#### Reason of non-use of ACT

In this study, we observed that lack of cash was cited but was not the main barrier to the using ACT. Other handicaps like "Not familiar with the use of ACT", "Absence of prescription in the HC", "Unavailability of ACT in the HC and with CHW" and the “Use of paracetamol” were potentially great barriers, especially in the south, to the use of ACT. In accordance to our findings, other studies have already highlighted that the use of anti-malarial drugs is prevented by: i) unregulated informal drug sales [55], stock-outs of ACT in the formal sale agencies (HC, CHW) [56–58], iii) and the practice of self-treatment [19, 59]. This means that even if malaria diagnosis was accessible and ACT becomes free, big challenges would remain to achieve the efficiency of this strategy. The proximity to the place of sale and the availability of ACT in this place of sales represents the global reason for non-use of ACT. ACT should be available at locations closer to beneficiaries and at the point of seeking for care (HC or CHW) in order to be used more frequently. According to our results, CHW was not the initial source of care seeking but had a role in the point of sale of ACT (58%). In Ghana, the Global Fund AMFm program increased ACT affordability, ACT availability, and ACT use [60].

### Factors associated with the use of ACT

A review of program and project evaluations regarding on-going interventions in BCC for malaria case management in other African countries found its effectiveness dependent on some limitations. In 2012–2014, Ethiopia’s Malaria Operational Plans (MOPs) evaluated BCC interventions on malaria and found that the adherence to AL treatment among malaria-infected patients in the Tigray region was significantly associated with three variables: radio ownership, belief that malaria cannot be treated traditionally and a delay in treatment seeking. The study recommended the use of radio as a channel to promote health messages regarding the importance of AL adherence and advocated for health extension workers to carry out specific trainings on communicating treatment instructions [61–63]. To increase adoption of key family health practices in Rwanda, the Kabeho Mwana project used community mobilization, social behaviour change, care groups and improved counselling. This project focused specifically, on the capacity building and mobilization of CHWs, care groups, local authorities, religious leaders and opinion leaders to educate families on important health practices. The project used care groups as the main foci for community mobilization and BCC. As results, fever management indicators increased: i) appropriate care-seeking for fever in the six districts reached to 75% and ii) appropriate treatment of fever increased from 20% in 2006 to 43% in 2011 [62]. In Senegal, successes in the communication and social mobilization components were also documented [64]. Nevertheless, the content of information centred on the recognition of danger signs and encouraging families to seek care early as possible was considered not sufficient enough in BCC campaigns. Globally, supply chain of BCC materials was almost discontinued, and BCC materials were not supplied in accordance with the needs in terms of quantity and quantity [62].

In both health districts, the OKT and DCO, an important proportion of caregivers stated they received information on the use of ACT, nevertheless ACT usage remained low. Some questions need to be answered. What was the form and the content of this information provided to caregivers? What type of information is the population able to understand? These questions need to be clearly answered in order to fully develop an approach to malaria eradication. Some studies, conducted through CHW, showed that their quality and type of training influences their performance and improves the quality of care delivered to the community, especially in an IMCI context [16, 65]. In the present study, mass media (radio and television) were weakly cited as sources of information whereas mass media was described to give some effectiveness of BCC on child survival, including malaria [66, 67]. Radio, television, newspapers, social networks and other channels related to lifestyle need to be considered in Benin’s BCC strategy to obtain a better level of control for malaria and other childhood illnesses. It will be helpful to clearly know for each region (cultural unit for example), what worked best in BCC for malaria case management and what BCC foci should be in the design of future malaria case management in Benin.

### Comparison between southern (OKT) and northern (DCO) districts

The differences between the two health districts need to be considered in order to address when necessary specific and specific interventions. The use of ACT was higher in the north than in the south. In contrast, the use of other anti-malarial drugs and herbal teas were higher in the south. However, the overall correct use of ACT remained low in both districts. The lack of money, as a reason of non-use of ACT, was the same in the OKT and DCO area. In the OKT, preference of paracetamol, unavailability of ACT at HC and in CHW, light fever, and forgetting the existence of ACT were the highlighted reasons for not using ACT treatment. In contrast, in the DOC area, most caregivers declared that they were not familiar with the use of ACT and that ACT was not prescribed at the HC. Even if the study was conducted in two different epidemiological areas, the results suggested that, BCC needs to be addressed to caregivers on the choice of anti-malarial drugs and the specific limitations of herbal tea and paracetamol in malaria treatment.

### Study limitation

The major limitation of this study is the overestimation of declared febrile children as malaria diagnosis was not simultaneously done. However, with a high self-medication, it is difficult to conclude the over or underestimation of malaria cases. There are possible limitations to the accuracy of the measurement of “use of ACT”, and “correct use of ACT” based on caregiver responses. However, the caregivers were solicited over a short two-week period of recall to help increase accuracy. Nevertheless, the perception of fever by caregivers may have influenced their search for care and/or the first treatment given. This consideration could lead to an underestimation of ACT use. However, this data was collected under real conditions of drug use and these inevitable parameters must be considered consistently in this type of study.

## Conclusion

In Benin, the use and the correct use of ACT for febrile children remains low. The major reason of not using ACT was that the caregivers were not familiar to the use of ACT, the unavailability of ACT in the HC and with CHW, the use of other treatment or anti-malarial medication not recommended by NMCP/WHO in the north and in the south. The action should be focused on enhancing the knowledge on ACT treatment, the availability of ACT drug in the HC and with CHW and, the prescription of ACT by health worker.

The study showed that having received information about the use of ACT is positively associated with the use of ACT. This point highlights the fact that efforts may not have been sufficiently integrated with social communication, which should be based on the behavioural determinants of populations, especially mothers on the front line, to take care of children.

## Additional files

Additional file 1: Benin map showing the study area. The study was carried out in two health districts. Ouidah-Kpomassè-Tori Bossito in the south and Djougou-Coparco-Ouaké in the north [23]. (PDF 350 kb)

Additional file 2: Reason of no treatment with ACT when fever in OKT and DCO health districts, Benin, 2011. Reasons of no treatment with ACT when fever, was presented according to health district: OKT and DCO. Dark bar corresponded to OKT and grey bar corresponded to DCO. (PDF 192 kb)

## Abbreviations

ACT: Artemesinin-based Combination Therapy
AL: Artemeter-lumefantrine
AMFm: Affordable Medicines Facility – malaria
BCC: Behabiour change communication
CHW: Community Health Workers
C-IMCI: Community-based Integrated Management of Childhood Illness
CQ: Chloroquine
CTB: Coopération technique Belge
DCO: Djougou-Copargo-Ouaké
DHS: Demographic and Health Survey
GF: Global Fund
HC: Health Centre
IMCI: Integrated Management of Childhood Illness
INSAE: Institut Nationale de la Statistique Appliquée à l’Economie
MoH: Ministry of Health
MOP: Malaria Operational Plans
NMCP: National Malaria Control Program
OKT: Ouidah-Kpomassè-Tori Bossito
PRR: Prevalence Risk Ratios
USAID: US Agency for International Development
WHO: World Health Organization
