# Supplementary figures and images for: Low use of artemisinin-based combination therapy for febrile children under five and barriers to correct fever management in Benin: a decade after WHO recommendation
Source: BMC Public Health. 2018 Jan 22;18:168. doi: 10.1186/s12889-018-5077-6 (PMC5778640; doi:10.1186/s12889-018-5077-6)

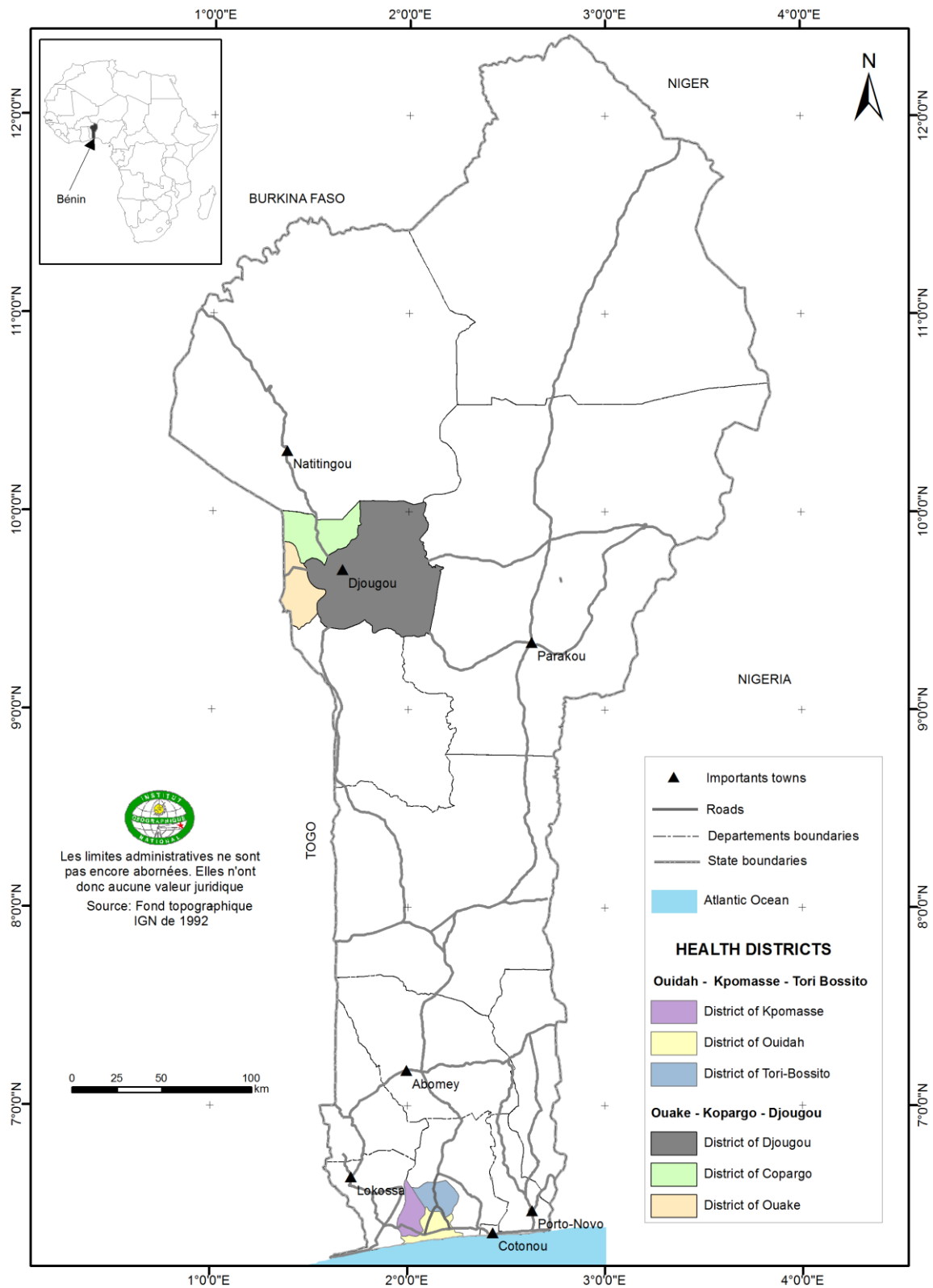

Supplement: Supplementary file 1 — Benin map showing the study area. The study was carried out in two health districts. Ouidah-Kpomassè-Tori Bossito in the south and Djougou-Coparco-Ouaké in the north [23]. (PDF 350 kb) [file 12889_2018_5077_MOESM1_ESM.pdf]

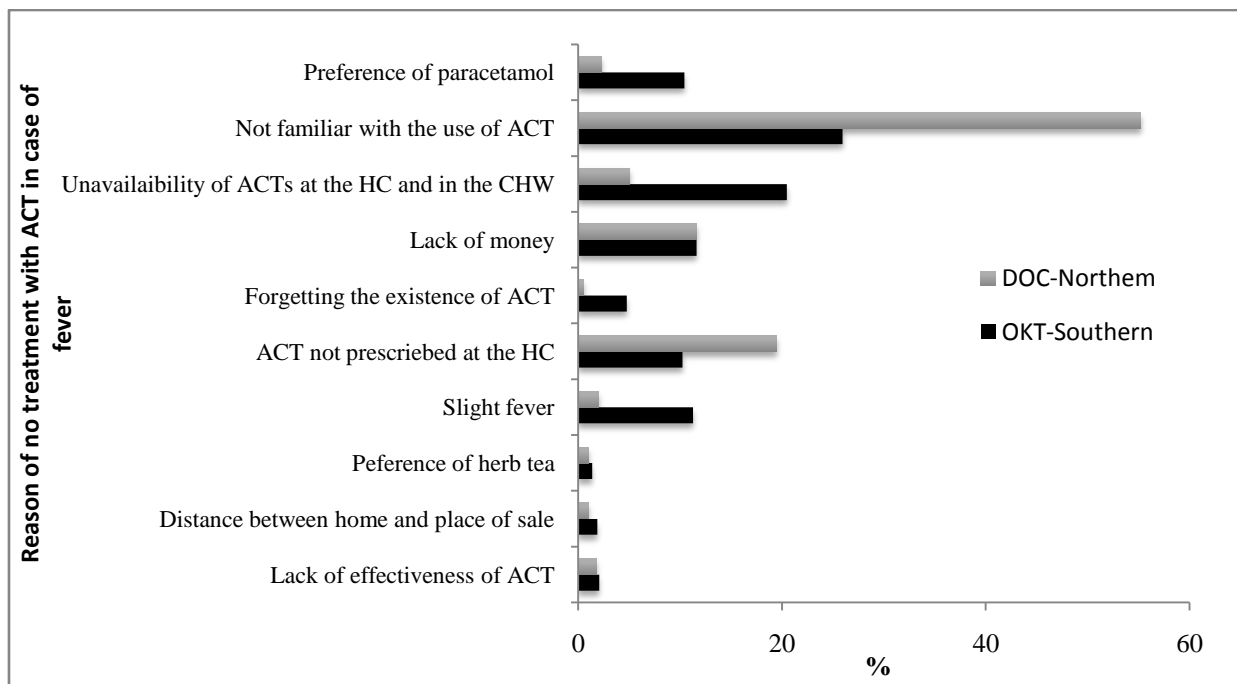

Supplement: Supplementary file 2 — Reason of no treatment with ACT when fever in OKT and DCO health districts, Benin, 2011. Reasons of no treatment with ACT when fever, was presented according to health district: OKT and DCO. Dark bar corresponded to OKT and grey bar corresponded to DCO. (PDF 192 kb) [file 12889_2018_5077_MOESM2_ESM.pdf]
